# Longitudinal study of the impact of the COVID-19 pandemic on diet and physical activity among Latinos of Mexican ancestry

**DOI:** 10.1186/s12967-024-05007-y

**Published:** 2024-04-09

**Authors:** Giovanna Muscogiuri, Lindsay Kohler, Oscar Parra, Lisa Soltani, Douglas Spegman, Dawn Coletta, Lawrence J. Mandarino

**Affiliations:** 1 Dipartimento di Medicina Clinica e Chirurgia, Unità di Endocrinologia, Diabetologia e Andrologia, Università degli Studi Di Napoli Federico II, Naples, Italy; 2 Cattedra Unesco “Educazione alla salute e allo sviluppo sostenibile”, University Federico II, Naples, Italy; 3 Division of Endocrinology, Department of Medicine, University of Arizona, Tucson, AZ USA; 4 Department of Health Promotion Sciences, College of Public Health, University of Arizona, Tucson, AZ USA; 5 Center for Disparities in Diabetes, Obesity, and Metabolism, University of Arizona Health Sciences, University of Arizona, Tucson, AZ USA; 6 El Rio Community Health Center, Tucson, AZ USA; 7 Department of Physiology, University of Arizona, Tucson, AZ USA; 8 Present Address: Pima County Department of Public Health, Tucson, AZ USA

**Keywords:** COVID-19, Type 2 diabetes, Diet, Food security, Physical activity

## Abstract

**Background:**

The COVID-19 pandemic caused societal disruption in the United States and most of the world, affecting many aspects of life, including healthcare and health-related behaviors such as diet, food security, and physical activity. Communities with economic and health disparities may have been particularly affected. This study was undertaken to determine how conditions in the early pandemic (January, 2021–February, 2022) affected Latino patients of Mexican Ancestry at high risk of type 2 diabetes mellitus who participated in *El Banco por Salud* biobank project in Tucson, Arizona.

**Methods:**

Baseline, prepandemic measurements were available in 17, 21, and 60 patients with normal hemoglobin A1c (HbA1c), prediabetes, and type 2 diabetes, respectively.

**Results:**

People with healthy HbA1c were significantly younger, less obese, and had higher HDL cholesterol. HbA1c was unaffected by the pandemic in any group. Triglycerides, total and HDL cholesterol levels fell in all groups during the pandemic. Physical activity levels in all groups were remarkably low, with most reporting no engagement in any voluntary physical activity. Engagement in physical activity or its enjoyment was lower in patients with diabetes and prediabetes than in younger, less obese patients. Major diet differences were between men and women and were present before the pandemic. Women consumed significantly more vegetables, fruit, and salad than men. The only pandemic-related change in diet was a drop in egg consumption, possibly explaining the fall in total cholesterol.

**Conclusion:**

Societal disruption during the COVID-19 pandemic had minimal effects on adverse health-related behaviors, cardiometabolic risk, or changes in glycemic control in a Latino community with diabetes and healthcare disparities in the Southwest US.

## Introduction

Coronavirus disease 2019 (COVID-19) is an infectious disease caused by a coronavirus (sars cov 2) that can have a profound detrimental impact on daily life [1]. To contain the spread of the COVID-19 pandemic, many governments put in place public health measures, such as lockdown conditions, masking, and social distancing. Consequently, people from many countries changed their habits, for example, having little or no options but to eat meals at home. In some countries and locales, people were required to adapt their physical exercise to indoor activity because they were not allowed to go to a gym or exercise in outdoor public places [2]. In addition, eating habits may also have been influenced by food insecurity resulting from a variety of causes, including household finances and supply chain problems [3]. Moreover, at times, access to healthcare was limited to virtual rather than face-to-face patient visits, especially for chronic conditions. Therefore, the pandemic itself, beyond any effects of COVID-19 infection, may have contributed to less healthy conditions.

It is evident that health professionals should also address the overall health status of COVID-19 survivors (post-COVID-19 syndrome), which is characterized by low-grade inflammation, malnutrition, and loss of fat-free mass [4]. In particular, the spread of COVID-19 has been shown to have an impact on the daily nutrition of adults worldwide [5]. Although a direct relationship between weight gain and dietary changes cannot be established, increased health benefits through increased snacking and increased daily food intake have been observed [6]. Results in children appear to lead to similar outcomes, as school cafeteria closures affected many families [7]. This situation also may have led to food shortages and insecurity in many low-income families where income could have been reduced by COVID-19. Unemployment payments and supplemental income may have compensated for their reduction in income from employment. Finally, for the elderly, people with obesity, and others with frailty, evidence has linked preventative measures that reduced interpersonal contact to changes in eating habits. These changes are associated with a decline in health [8]. Furthermore, it's crucial to note that obesity significantly impacts the immune response to infections and COVID-19 outcomes [9, 10]. Individuals with obesity are at higher risk of severe illness and complications from COVID-19 due to impaired immune function and chronic inflammation [9, 10].

The situation is even worse in developing countries, where the spread of the COVID-19 pandemic has reinforced the need to redefine “food security.” Although food supplies may be clearly "available", many developing countries do not have an adequate supply chain for food distribution and accessibility. For this reason, families may not be able buy enough food and therefore must change their eating habits, leading to deterioration in their health [11]. It is important to establish this dichotomy when discussing cultural changes during the COVID-19 pandemic, as food decisions are determined by many factors. To date, no studies have been conducted to understand the changes of nutritional habits and physical activity in Latino patients of Mexican Ancestry who are at high risk for type 2 diabetes mellitus and cardiovascular risk factors. In this study, we collected longitudinal data to characterize any changes in eating habits and physical activity among Latinos enrolled in the *El Banco por Salud* (El Banco) biobank [12] during the COVID 19 pandemic.

## Materials and methods

### Study sample

*El Banco por Salud* [12] is a biorepository created and launched in January 2018 by the University of Arizona Health Sciences Center for Disparities in Diabetes, Obesity, and Metabolism (CDDOM) in partnership with El Rio Community Health Center, a Federally Qualified Health Center, in Tucson, Arizona. All participants in the current study were recruited from active participants in *El Banco por Salud*. Inclusion and exclusion criteria were identical to El Banco [12], and this study was approved by the University of Arizona Institutional Review Board.

### Design

**Fig. 1:**
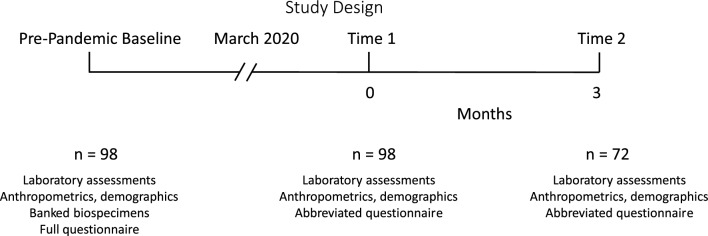
Design of the study. Patients enrolled in El Banco por Salud were originally enrolled and studied in 2018–2019, with baseline anthropometrics, clinical laboratory values, and questionnaire data collected at that time. Approximately 1 year after onset of pandemic condions, starting in January, 2021, patients were recontacted for the follow up study of the effects of the pandemic. At the first follow up visit, Time 1, patients had repeat anthropometric and laboratory measurements and completed an abbreviated questionnaire regarding diet and physical activity. Each patient was scheduled for a second follow up visit, Time 2, 3 months after their Time 1 visit. All the measurements at Time 1 were repeated at Time 2. All studies were completed by February, 2022. A total of 98 participants agreed to study at Time 1; 72 of these agreed to a second follow up visit at Time 2. All data were included in analysis by mixed linear model or logistic regression

Ninety-eight El Banco participants were recalled for enrollment in this study. There were 60 patients with confirmed type 2 diabetes mellitus, 21 who were deemed to have prediabetes by HbA1c (between 5.7 and 6.4%) and 17 participants with normal HbA1c levels (< 5.7%). This distribution approximately mirrors that of *El Banco por Salud*. Baseline, pre-COVID-19 diet and physical activity questionnaires, anthropometrics and laboratory values were determined as described [12]. These baseline studies were conducted approximately 0–2 years before the onset of the pandemic, which we consider to be March 2020 because local restrictions were put in place mainly in that month. The study design (Fig. 1) consisted of three longitudinal time points (each time point consisting of a virtual and in-person component): the initial, pre-COVID-19 study date, with two additional study dates separated by 3 months. The initial El Banco visit served as a pre-COVID-19 baseline comparison. Participants were asked to provide biospecimen samples for plasma, serum, lipid panel, metabolic panel, a shortened version of the original El Banco questionnaire and anthropometric measures including weight and height to determine body mass index (BMI). During the pandemic, initial contact of existing El Banco participants was completed by phone call. If El Banco participants agreed to participate in this follow-up study, they were scheduled for the initial study date visits during the pandemic. Participants then were sent an email with unique links to their specific electronic consent form and study questionnaire, through the REDcap database. The questionnaire link did not become active, until the participant completed and submitted the electronic consent form. Coordinators either were on the phone or logged in through Zoom Health to explain the consent form and confirm the volunteer’s comprehension of the study. Coordinators were also available for any questions while the participant completed the study questionnaire. Once this was completed, the participant was scheduled for an in-person visit, to provide biospecimen samples and anthropometric measurements. The anthropometric measurements were collected within seven days of completing the initial, virtual consent and questionnaire visit. A follow-up visit during the pandemic (Time 2, Fig. 1) was scheduled for 3 months ± 1 week after the first study during the pandemic (Time 1, Fig. 1). Thus, there were three study visits in all, a pre-pandemic baseline that was part of the original biobank study data, Time 1 (first study during pandemic, approximately 1.5–2.5 years after pre-pandemic baseline, and Time 2 (approximately 3 months after Time 1). All participants completed the study between January, 2021 and February, 2022.

### Diet assessment

Food security was assessed using the American Household Food Security Survey module [13–15]. Diet composition was estimated using validated surveys [16, 17]. The University of Arizona REDcap electronic data management system was used for data collection, security and management, and data transmission.

### Physical activity

Physical activity was evaluated by questionnaire, evaluating both an assessment of physical activity at work and voluntary physical activity asking energy frequency (heart rate), average and activity intensity for at least 15 min in a 7-day period [18].

### Statistical analysis

Descriptive statistics were produced for all participants. Dependence of continuous variables (e.g. HbA1c, plasma lipids) on the independent variables of time (baseline, Time 1 and Time 2), sex (F/M), and diabetes status (healthy, prediabetes, type 2 diabetes) was assessed using mixed model analysis (xtmixed command, Stata software v. 17.0, StataCorp LLC, College Station, TX). Ordinal dependent variables were assessed versus time (baseline, Time 1 and Time 2), sex, and diabetes status using ordinal logistic regression analysis (ologistic command, Stata). Binary dependent variables were assessed using logistic regression (logistic command, Stata). A *P* value of < 0.05 was considered significant.

## Results

Baseline and follow up anthropometric and cardiometabolic characteristics are given in Table 1 for all participants at baseline (pre-pandemic) and Times 1 and 2 during the COVID-19 pandemic (Table 1). As expected, all participants aged during the study period, and patients with healthy HbA1c levels were younger, had lower HbA1c, BMI and plasma triglycerides. Those with healthy HbA1c, however, had higher total and LDL cholesterol levels than patients with type 2 diabetes, which may have reflected higher levels of treatment in patients that had been identified with abnormal glucose tolerance or diabetes especially since participants were recruited from a health center where they more likely to receive guideline-directed management including statins for patients with diabetes. Total cholesterol levels fell in all participants during the pandemic. Patients with type 2 diabetes had the lowest HDL cholesterol levels (controlled for sex), and HDL cholesterol fell throughout the pandemic. HbA1c did not change in any group during the period of the pandemic covered by this study.

**Table 1:** Age, BMI, plasma lipids and HbA1c levels at baseline and at time 1 and 2 according to diabetes status

	Normal glucose tolerance			Pre-diabetes			Type 2 diabetes mellitus		
	Baseline pre-COVID-19	Pandemic time 1	Pandemic time 2	Baseline pre-COVID-19	Pandemic time 1	Pandemic time 2	Baseline pre-COVID-19	Pandemic time 1	Pandemic time 2
N (F/M)	17 (11/6)	17 (11/6)	8 (6/2)	21 (17/4)	21 (17/4)	16 (14/2)	60 (42/18)	60 (42/18)	48 (33/15)
Age (years)**^††^	42 ± 15	45 ± 15	45 ± 15	53 ± 13	55 ± 13	55 ± 12	54 ± 12	56 ± 12	57 ± 11
BMI (kg/m^2^)**	28 ± 4	28 ± 4	28 ± 3	33 ± 7	33 ± 8	32 ± 9	34 ± 7	34 ± 7	35 ± 8
HbA1c (%)**	5 ± 0	5 ± 0	5 ± 0	6 ± 0	6 ± 0	6 ± 1	9 ± 2	8 ± 2	8 ± 2
Triglycerides (mg/dL)	155 ± 144	118 ± 83	96 ± 28	191 ± 195	178 ± 118	148 ± 72	186 ± 98	181 ± 88	198 ± 102
Total Cholesterol (mg/dL)**^††^	195 ± 42	182 ± 46	175 ± 53	195 ± 31	178 ± 39	177 ± 38	172 ± 47	157 ± 41	163 ± 41
HDL Cholesterol (mg/dL)**	53 ± 13	49 ± 13	44 ± 9	53 ± 10	48 ± 11	51 ± 14	45 ± 11	43 ± 12	43 ± 14
LDL Cholesterol (mg/dL)**	115 ± 35	110 ± 42	112 ± 7	109 ± 30	96 ± 39	98 ± 48	88 ± 32	77 ± 30	84 ± 29
VLDL Cholesterol (mg/dL)	31 ± 29	24 ± 17	19 ± 6	38 ± 39	36 ± 24	30 ± 14	37 ± 20	36 ± 18	40 ± 20
LDL/HDL	2 ± 1	2 ± 1	3 ± 1	2 ± 1	2 ± 2	2 ± 1	2 ± 1	2 ± 1	2 ± 2

Data are given as Mean ± STD. Analysis of changes over time were performed using a mixed model linear regression controlling for diabetes status

**P < 0.01 for diabetes status

^††^*P* < 0.01 for time. See Fig. 1 for Study Design defining Times 1 and 2

### Effect of the COVID-19 pandemic on diet

The results of answers to self-reported questions regarding eating habits of the participants are given in Table 2. Analysis was conducted using ordinal logistic regression over time, controlled for sex and diabetes status. The pandemic significantly decreased reported consumption of eggs, independent of other variables. Otherwise, there were no significant effects of the pandemic (“time” variable) on diet. However, other patterns emerged. Men reported being significantly more likely to consume flour tortillas, refried beans, hamburgers or cheeseburgers, fried potatoes, fried chicken, tacos/burritos/enchiladas, other dishes with meat, pizza, fruit juice, any potatoes, roast pork/beef/steak. Women reported consuming more green salads and vegetables.

**Table 2:** Effect of the COVID-19 pandemic on diet

Question	Overall model *P*	Regression coefficients (β)		
		Time	Sex	Diabetes status
Flour tortillas	< 0.01**	− 0.25	0.85**	0.38*
Refried beans	< 0.01**	− 0.26	0.98**	0.13
Hamburgers/cheeseburgers	< 0.01**	− 0.96	0.92**	− 0.20
Green salad	< 0.01**	− 0.11	− 0.89**	− 0.10
French fried/fried potatoes	< 0.01**	− 0.22	0.73**	0.17
Fresh vegetables	< 0.01**	− 0.26	− 0.73**	− 0.07
Fried chicken	0.01**	− 0.05	0.85**	0.03
Eggs	0.01**	− 0.37*	0.46	− 0.14
Tacos/burritos/enchiladas	0.01*	− 0.08	0.75**	0.12
Other mixed dishes with meat	0.02*	− 0.20	0.68**	0.07
Pizza	0.03*	0.04	0.51*	− 0.38*
Fruit juice	0.04*	− 0.18	0.62*	− 0.11
Any potatoes	0.05*	− 0.15	0.68**	0.12
Roast pork, beef, or steak	0.05*	0.07	0.60*	− 0.22
Cheese/cheese spread	0.06	− 0.10	0.39	− 0.28
Fruit (fresh/frozen/canned)	0.08	− 0.21	− 0.22	− 0.25
Cake, sweet rolls, doughnuts	0.13	− 0.03	0.49	− 0.20
Use fat or oil to fry, cook, or season	0.16	− 0.10	0.52	0.10
Tomatoes/Fresh salsa	0.30	− 0.26	− 0.10	0.04
Salad dressing	0.40	− 0.11	− 0.09	− 0.21
Potato/corn chips, peanuts	0.50	0.18	0.08	− 0.03
Vegetable stew/soup	0.51	− 0.21	− 0.01	0.06
Whole milk	0.62	− 0.11	0.26	0.01

Participants were asked: “Think about your eating habits over the past month or so. About how often do you eat each of the following foods either at home or in restaurants?” Shown are overall model significance and regression coefficients for time, sex, and BMI. Analysis was performed using ordinal logistic regression, controlling for sex and BMI. Females were coded “0” and males were coded “1”. Codes for diabetes status were normal HbA1c, “0”: prediabetes. “1”; and diabetes “2”

*P < 0.05, **P < 0.01

### Effect of the COVID-19 pandemic on food security

The pandemic appeared to have little effect on food security in these participants (Table 3). Patients with diabetes or prediabetes were marginally more likely to report having insufficient funds to eat balanced meals.

**Table 3:** Effect of pandemic food security

Question	Overall model *P*	Regression coefficients (β)		
		Time	Sex	Diabetes status
Cut the size of or skip meals	0.11	− 0.57	− 0.15	0.27
Eat less because you did not enough money	0.32	− 0.30	− 0.79	0.13
Go hungry because you did not enough money	0.33	− 0.40	− 1.00	0.17
Feel like you couldn’t afford to eat balanced meals	0.07	− 0.28	− 0.24	0.17*

Participants were asked, “In the last 12 months, did you…”. Shown are overall model significance and regression coefficients for time, sex, and BMI. Analysis was performed using ordinal logistic regression, controlling for sex and BMI. Females were coded “0” and males were coded “1”. Codes for diabetes status were normal HbA1c, “0”: prediabetes. “1”; and diabetes “2”

*P < 0.05

### Effect of the COVID-19 pandemic on physical activity

**Fig. 2:**
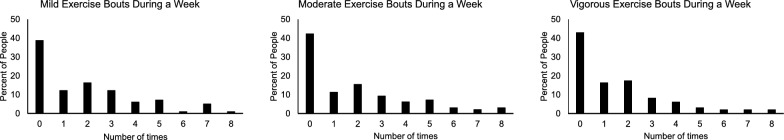
Voluntary physical activity before the pandemic in participants of this study. Participants were asked, during the period of a week, “How many times do you engage in mild, moderate, or vigorous activity for at least 15 min?” Examples of the three exercise intensities were given to guide answers. Overall, there were no changes in voluntary physical activity during the pandemic (not shown)

With regard to voluntary physical activity, participants were asked on a scale of 1 to 8 or more how many times per week they engaged in light, moderate, or vigorous exercise for at least 15 min per “bout” of physical activity. Examples were given of types of exercise in the three categories. Even at baseline, the vast majority of participants reported very little physical activity, with most participants reporting that they did zero or one voluntary exercise bout per week at any intensity for at least 15 min. This is shown for the pre-COVID-19 baseline in Fig. 2.

Self-reported physical activity related to work or voluntary exercise was also assessed by questionnaire before and during the COVID-19 pandemic (Table 4). Regarding work related physical activity, participants were asked how much of their workday was spent sitting, standing, or walking on a scale of 1–5. Most participants reported walking or standing versus sitting, but there was no effect of the pandemic on these habits. Men reported walking significantly more on the job than women. Participants also were asked how much of their workday was spent in light or heavy manual labor. Participation in light manual labor was unrelated to the pandemic, sex, or diabetes status. However, men reported spending much more time engaged in heavy manual labor at work (*P* < 0.001), with no effect of the pandemic or diabetes status. With regard to the number of voluntary light, moderate, or vigorous exercise bouts per week lasting at least 15 min, the pandemic and sex had no effect. However, patients with prediabetes or type 2 diabetes reported significantly less voluntary exercise bouts at any intensity. There was a significant effect of the pandemic on participants reporting they engaged in less physical activity because people said they should, but participants with diabetes or prediabetes reported being more likely to exercise because of recommendations. On the other hand, although the pandemic and sex did not affect whether people report enjoying exercise or feeling restless if they do not exercise, patients with diabetes or prediabetes were less likely to enjoy exercise or feel restless in its absence.

**Table 4:** Effect of pandemic on physical activity

Question	Overall model *P*	Regression coefficients (β)		
		Time	Sex	Diabetes status
How much of your job involves sitting?	0.08	0.18	− 0.46	0.18
How much of your job involves standing?	0.24	− 0.02	0.49	− 0.11
How much of your job involves walking?	0.05*	− 0.15	0.64*	− 0.10
How much of your job involves light manual labor?	0.04*	− 0.09	0.28	− 0.39
How much of your job involves heavy manual labor?	< 0.01**	− 0.12	1.42**	− 0.10
Light exercise bouts per week lasting at least 15 min?	0.09	0.14	0.36	− 0.29
Moderate exercise bouts per week lasting at least 15 min?	0.11	0.09	0.29	− 0.32*
Vigorous exercise bouts per week lasting at least 15 min?	0.01*	− 0.07	0.20	− 0.48**
To what extent do I exercise because people say I should?	< 0.01**	− 0.35*	− 0.15	0.57**
To what extent do I enjoy my exercise?	0.08	0.06	− 0.02	− 0.38*
To what extent do I feel restless if I don’t exercise?	0.06	− 0.05	0.27	− 0.37*

Shown are overall model significance and regression coefficients for time, sex, and BMI. Analysis was performed using ordinal logistic regression, controlling for sex and BMI. Females were coded “0” and males were coded “1”. Codes for diabetes status were normal HbA1c, “0”: prediabetes. “1”; and diabetes “2”

*P < 0.05, **P < 0.01

## Discussion

This observational, longitudinal study was undertaken to better understand how changes in health-related behaviors during a societal disruption such as that caused by the COVID-19 pandemic might alter cardiometabolic risk in Latinos of Mexican Ancestry who live in the border region of the Southwest United States, in Tucson, Arizona. Since members of this community are at elevated risk of type 2 diabetes, we recruited participants in *El Banco por Salud*, a Latino diabetes biobank focused on type 2 diabetes and cardiometabolic risk [12]. Genetically, such individuals on average have a 45% contribution of genes derived from Indigenous American ancestry on average, with the remainder primarily being European ancestry (unpublished results). Participants were either healthy or had prediabetes or type 2 diabetes mellitus, defined according to criteria of the American Diabetes Association based on HbA1c [19]. The present study focused on the impact of voluntary or forced changes in health-related habits during the pandemic, rather than COVID-19 infection itself. This longitudinal study provides the first evidence on the changes of nutritional habits and physical activity in Latino patients of Mexican Ancestry who are at high risk for type 2 diabetes mellitus during the COVID-19 pandemic.

Regarding anthropometric and clinical characteristics of the participants at the pre-pandemic, baseline timepoint, participants with healthy HbA1c levels were younger, had lower HbA1c, BMI, and plasma triglycerides but had higher total and LDL cholesterol levels. This could be due to the fact that subjects with abnormal glucose tolerance also usually receive cholesterol lowering medications in order to reach therapeutical targets for patients with diabetes. Patients with type 2 diabetes had the lowest HDL cholesterol levels (controlled for sex). A major finding of this study is that HbA1c did not change in any group during the period of the pandemic covered by this study, indicating that diabetes care likely was not affected to the point that HbA1c was altered. Since a time period of 3 months elapsed during the period of study that fell within the pandemic, major changes in glucose control in patients with type 2 diabetes likely would have been detected. This finding suggests that well-managed Community Health Centers maintained their usual level of diabetes care despite the problems in healthcare delivery during this initial part of the pandemic when uncertainties resulted in lockdowns and a fall in patient visits. To mitigate this, El Rio Community Health Center engaged in systematic telephonic outreach to patients with elevated risk for complications (including patients with diabetes) to ensure they were taking medications, had refills and to offer telemedicine visits, making over 40,000 outreach telephone calls to our patients in the first year of the pandemic. The COVID-19 pandemic has been associated with an increased risk of developing or worsening cardiometabolic risk factors [20] that have been ascribed to several factors. First, there was a marked decline in hospital-based clinic visits for non-COVID-related conditions and this was due to patient fear of contracting COVID-19, public health messages to avoid the outpatient clinic for non-COVID-19 related conditions, and limited access to emergency medical services (because of reduced staffing from illness or isolation requirements) [21–23]. This also resulted in a reduction of routine laboratory testing that is an important part of cardiovascular risk reduction, since medical treatment for diabetes, hypertension and dyslipidemia involves the routine measurement of laboratory parameters, both for safety and for appropriate modification of therapies. In addition, the loss of employment, for many, may have resulted in the loss of insurance benefits and thus difficulties affording glucose-lowering, antihypertensive and lipid-lowering medications [24]. However, because many patients in El Banco por Salud have insurance coverage through Medicaid or other needs-based programs, this may not have been a larger problem during the pandemic than what is usually experienced by these patients. Nevertheless, total cholesterol levels fell in all participants during the pandemic. This may have been partially due to dietary changes during the pandemic (see below). Perhaps counterbalancing this potential cardioprotective change, HDL levels also declined. This could be related to physical activity changes that are described below.

The present study revealed a number of sex differences in diet that were present before the pandemic and were sustained during the pandemic period. Men reported being significantly more likely than women to consume flour tortillas, refried beans, hamburgers or cheeseburgers, fried potatoes, fried chicken, tacos/burritos/enchiladas, other dishes with meat, pizza, fruit juice, any potatoes, roast pork/beef/steak. On the other hand, women reported consuming more green salads and vegetables than men. Sex hormones have been reported to have an impact on eating behavior, in the so-called “homeostatic” control of energy intake as well as “the hedonic” control of food intake [25, 26]. Our finding is in agreement with the current evidence in literature. A recent study by Stea et al. investigating the sex-based differences in food choices with a cross-sectional study in 21 European countries (n = 37,672 individuals) found that women (n = 19,815) were more prone to have a higher consumption of fruit and vegetables than men (n = 17,857) [27]. In line with these results, the study by Wardle et al. in 19,298 individuals (8482 men and 10,816 women) highlighted that fruit and fiber intake was higher in women than in men that preferred to consume more high-fat foods and salt [28]. The results of the current study extend those findings to individuals from an unrelated population, Southwest U.S. Latinos of Mexican Ancestry. The present findings indicate that there were few changes in reported diet during the first year of the pandemic. However, LDL cholesterol levels fell in all participants during the pandemic. This could be explained [29] by the fact that egg consumption fell significantly during 2020. Regarding food security, there were no significant changes in food security during the pandemic, which is consistent with the lack of major changes in reported diet described above. There was a marginally significant increase in patients with type 2 diabetes in their reported ability to afford to eat balanced meals.

Regarding physical activity, there were sex and diabetes status-related differences in self-reported voluntary and job-related physical activity before the pandemic. Men reported engaging in more job-related walking and heavy manual labor, consistent with individuals with lower economic resources and education level attainment, characteristic of *El Banco por Salud* [12]. Most striking, however, was the overall low level of voluntary physical activity, with the vast majority of participants reporting little to no voluntary activity lasting at least 15 min every week. In particular, participants with prediabetes and type 2 diabetes reported significantly lower levels of moderate and vigorous voluntary exercise, even below the already low levels of activity in individuals with “healthy” HbA1c levels. In this regard, it is worth noting that participants with lower HbA1c were also overweight, while patients with prediabetes and diabetes on average suffered from obesity. Low levels of physical activity are consistent with obesity. Interestingly, when participants were asked about their feelings regarding physical activity, during the pandemic people overall were less likely to report that they exercised because they were told to do so, but patients with diabetes had the opposite effect, where independent of the pandemic or sex they were more likely to engage in exercise because they were told to do so. This may have been a result of clinical recommendations to these patients. Moreover, independent of the pandemic or sex, participants with diabetes or prediabetes were less likely to report they enjoyed exercise or felt restless if they refrained from exercise. A portion of the decline in HDL during the pandemic may have been attributable to lower physical activity levels, but this is not entirely clear from these data.

This study had several limitations. First, the sample size, which was only 98 for the first assessment, fell due to difficulties in recontacting patients during the pandemic. If those patients who did not participate at the second time point were somehow different from those who did participate, this may have affected the results. Statistical techniques used account for dropout but assume this is random. Second, since COVID-19 infection was not assessed and participants did not always know if they had been affected, it is not possible to dissect the effect of pandemic conditions of hardships from those of COVID-19 infection. In addition, because diet, physical activity and food security depended on self-reported measures, these variables have inherent limitations. In fact, although our study primarily focuses on voluntary and work-related physical activity due to data availability, we acknowledge that this decision may have constrained our comprehensive assessment of physical activity patterns during the pandemic. Finally, it is important to note that while the study focused on the impact of changes in health-related habits during the pandemic, rather than COVID-19 infection itself, the potential role of factors such as vitamin D intake in influencing immune response and COVID-19 outcomes should also be considered [30].

In conclusion, the findings of this study describe information regarding COVID-19 pandemic-related changes in eating habits and physical activity and their consequences on cardiometabolic risk factors in Latino patients of Mexican Ancestry who are at high risk for type 2 diabetes mellitus. Although analysis of the data revealed a community with deficits in physical activity and food security, there were surprisingly few pandemic-induced changes in these parameters or cardiometabolic risk factors that may be connected to health behaviors. Major differences in the diets of men and women suggest that behavioral interventions in diet and physical activity should be not only culturally appropriate but designed to have a sex-specific component.

## Data Availability

The datasets used and/or analysed during the current study are available from the corresponding author on reasonable request.
